# Folie à Deux and its interaction with early life stress: a case report

**DOI:** 10.1186/s13256-016-1128-8

**Published:** 2016-12-01

**Authors:** Alessandra Vargas Alves Nunes, Sandra Odebrecht Vargas Nunes, Talita Strano, Gilberto Pascolat, Gustavo Manoel Schier Doria, Mauricio Nasser Ehlke

**Affiliations:** 1 Pediatrics Department, Hospital Universitário Evangélico de Curitiba, 2885 Padre Agostinho, apto 1304 torre barigui, ZIP 80710-000 Curitiba, Paraná Brazil; 2 Department of Health Science, Universidade Estadual de Londrina, Londrina, PR Brazil; 3 Psychiatry Department, Universidade Federal do Paraná, Curitiba, PR Brazil

**Keywords:** Case report, Early psychosis, Child abuse, Child development, Induced delusional disorder, Shared paranoid disorder, Folie à deux

## Abstract

**Background:**

Folie à deux is a very rare psychiatric syndrome in which a psychotic symptom is transmitted from one individual to another. We present a case of folie à deux occurring during childhood, which is not an usual presentation of this syndrome. In this case, the disorder is correlated with child abuse and neglect, which possibly had a role in the development of the symptoms in our case.

**Case presentation:**

We present a case of folie à deux between an “induced” 9-year-old black Brazilian boy and the “inducer”, his grandmother. They were found to be sharing similar auditory and visual hallucinations and delusional beliefs. The boy was neglected by his parents and was being cared for by his grandmother, who had a history of mental disorder.

**Conclusions:**

The close relationship between the boy and his grandmother, the family history of first-degree psychosis, and the child abuse and neglect suffered by the boy may have altered his vulnerability to early-onset psychosis and, in this case, folie à deux*.*

## Background

The existence of early-onset psychosis in children has been questioned owing to the difficulty in differentiating a psychotic disorder and a childhood fantasy as part of normal development. However, childhood psychotic disorders are now well-known entities; their clinical presentation in childhood differs from the adult form, but both are considered to have the same pathology [1].

Child abuse and neglect by parents and other caregivers can be considered a risk factor for psychotic disorders [2] owing to the biological mechanisms and the activation of cortisol by the hypothalamic–pituitary–adrenal axis [3]. In addition, gene–environment interactions are likely to play a role in the relationship between childhood trauma and psychosis. In a recent study, the relationship between childhood abuse and psychosis was moderated by the BDNFVal66Met polymorphism, whereby an individual who carries the Met allele has a higher risk of developing psychotic experiences when exposed to childhood abuse compared to individuals who carry the Val allele [4].

Child abuse and neglect is a complex experience that includes emotional, physical, and sexual abuse, as well as emotional and physical neglect. This childhood adversity can be associated with future mental disorder [5]. Because child abuse occurs during the critical formative period in which the brain is being physically sculpted, the impact of extreme stress can leave an indelible mark on the brain’s structure and function. Such abuses seem to induce a cascade of molecular and neurobiological effects that irreversibly alter neuronal development, enabling the individual to develop psychiatric symptoms such as psychosis, aggression, and anxiety, and even influencing the way they perceive the environment and the people around them [6].

The theory of traumatology development, which assesses the impact of adverse events in early physical and psychological development, shows that early life trauma leads to lasting changes in the developing brain. These changes can alter neurotransmitters and hormones that modulate development of the neuronal migration process as well as the differentiation and the synaptic proliferation that can affect the brain in development [7].

In this context, it is possible for individuals to develop a shared psychotic disorder, also known as folie à deux. This is a very rare psychiatric syndrome in which a psychotic symptom is induced in one individual by another [8]. Its main feature is a delusion that develops in an individual who is involved in a close relationship with the “inducer” or “primary case,” who already has a psychotic disorder with prominent delusions. The syndrome is called a shared psychotic disorder in the Diagnostic and Statistical Manual of Mental Disorders fourth edition (DSM-IV), but it is not present in the current DSM-5 [9]. In the 10th edition of the International Classification of Disease, the disorder is called induced psychotic disorder [10].

In shared psychosis, the individual who is influenced by the inducer is someone who possibly has low intelligence. They are also shy, passive, and have lower self-esteem than the inducer, often living in a constant relationship with the latter and almost completely isolated from the rest of the world, and unrelated to a network of social contacts [8].

Schizophrenia is probably the most common diagnosis of the primary case, although other diagnoses may include delusional disorder or mood disorder with psychotic features. The content of the shared delusional beliefs may depend on the diagnosis of the primary case and can include bizarre delusions, delusions congruent with the mood, or non-bizarre delusions characteristic of delusional disorder [9].

In reporting this case, we aim to associate early-onset psychosis with childhood adversities, as well as with the family environment and a history of mental disorders, which may also trigger the beginning of a shared psychotic disorder.

## Case presentation

A 9-year-old black Brazilian boy, accompanied by his grandmother, was referred to our hospital by the Emergency Unit owing to auditory and visual hallucinations. Because of his increasingly aggressive behavior at school, our patient was being treated in a Basic Health Unit near his home. The prescribed treatment was carbamazepine at night, which resulted in partial control of his symptoms. However, at the time of admission, he had stopped taking the medication for 2 days, causing the visual hallucinations to return. He had no fever or other changes in this period and denied the use of drugs or other illegal substances, except prescribed haloperidol and promethazine. When he arrived at our hospital he was calm and had no other complaints.

His treatment started approximately 2 years prior to the current admission when he started hearing voices that commanded him not to have friends and to kill himself. Such hallucinations always occurred at home, at around 6 p.m., and included visual perceptions of distinct characters. These visions often took the shape of bloody human beings: one was black, one was a baby, and some could change shape (get fatter or thinner, or become larger or smaller). The hallucinations were perceived as a meeting but our patient was unable to understand the language spoken. At the end of this meeting, one of the characters would tell him that he should kill himself. He was convinced that they were talking about him and sending him a special message. Our patient reported sleep deprivation due to these hallucinations.

According to his grandmother, our patient had bizarre behaviors, such as writing on the wall, throwing things on the ground, littering the closet, and walking around with a knife, without being aware of them soon after the event. She also reported recent aggressive behavior around his school colleagues and daily mood swings.

Our patient denied thoughts of worthlessness, anhedonia, or episodes of fast thinking and euphoria. He did not report a history of head trauma, seizures, or other medical conditions that could cause psychosis.

Our patient’s father, mother, maternal grandparents, two uncles, and two half-brothers from the same father had a history of mental disorders. Our patient lived with his grandmother, his mother, and two younger siblings. His parents were not caregivers, nor did they provide proper supervision. His father, who did not live in the same house, had attempted suicide three times; at this time he was functionally impaired and supported by a government program. His mother, although living in the same house, was not his caregiver nor had any responsibility for his siblings. Only his grandmother showed any characteristics of caregiving. According to his grandmother, our patient attended school without cognitive impairment and was in fifth grade. He had never failed a school year.

His physical examination was unremarkable, with only warts found on his hand and nose. The warts led us to suspect sexual abuse, which was later discarded owing to the diagnosis of common warts, the absence of lesions in the genital region, and our patient’s denial of a history of abuse. Laboratory tests, electroencephalogram and neuroimaging were performed on our patient according to standard protocol in such cases, which showed no abnormalities.

Our patient was hospitalized and prescribed risperidone. During this period, he recovered from his symptoms. Following advice from our psychiatric department, our patient was discharged after 3 days and prescribed 1 mg of risperidone per day. Our psychiatric department requested to talk with our patient and his grandmother separately, because of a suspicion that the grandmother was influencing our patient’s responses and auditory and visual hallucinations.

Our patient and his grandmother returned 15 days later and were interviewed separately. Our patient reported that the symptoms improved after he started taking the drug, but said that he felt more isolated now because his schoolmates began to call him crazy. He also reported that his father, who has alcoholism, is an Indian and has no contact with him because he lives with an Indian tribe in the woods. He said he hardly sees his mother because she is never home and believed to be working. He reported that he spends most of the time at home with his grandmother and two younger sisters. When questioned about his relationship with his grandmother, he said they get along very well and that she is always telling a story about a haunted house where she had lived before he was born, and where people could see shadows, bloody murders, and ghosts, and hear voices telling them to kill themselves.

During the interview with the grandmother, she stated that she has a kind of “super power” that allows her to sense everything that is going on with the people in her family, including the murder of some family members, and that all family members are aware of her power and admire it. She did not have stable work, spent most of her time at home, and showed great affection for the patient. She reported a haunted house, where she used to live 15 years ago with her family and where she saw ghosts and heard voices. When asked about more details of those events, she said that objects in the house would break for no reason, and that it was possible to see bloody people changing shape, becoming fatter or thinner. Among those people there was a black man, a baby, and a woman who would tell her to kill herself. We asked if those were visions similar to her grandson’s hallucinations and she said they were and that even though he has never lived in that house, he has had similar visions. She added that by the age of 2 years he had already had those same visions, which disappeared for a time but returned in the past 2 years.

According to their social worker, our patient had a good academic performance and had been monitored since 2008 by the Child Protection Council, owing to the fact that he was abandoned by his mother, had an alcoholic father, and was being raised by his grandmother.

The hypothesis of a shared psychotic disorder, also known as folie à deux, was raised based on the similarity of the delusions and hallucinations of the boy and the grandmother and their close relationship. A mental status examination did not show enough psychopathological criteria to support any other differential diagnoses, such as (1) schizophrenia, because the diagnosis requires cognitive and social dysfunction and a careful clinical follow-up, which was not seen in this case; (2) bipolar disorders, because patients have psychotic symptoms during episodes of mania or depression, but in this case our patient did not show any prominent affective or mood symptoms, which are essential to establish the diagnosis; (3) other psychoses due to general medical disease, which were excluded because results from physical and complementary examinations were normal; 4) mental retardation or developmental disorders, which were excluded owing to the lack of cognitive or language deficit; and (5) substance abuse, because there were no signs of substance abuse in this case.

## Discussion

This report illustrates the case of a boy who experienced childhood trauma and developed early-onset psychosis. Our patient had a family history of mental disorders and was neglected by his first-degree relatives: mother and father. Our findings suggest the hypothesis that early-onset psychosis is related to negative life events during childhood, such as (1) neglect, that is, lack of an affective relationship with caregivers; and (2) frightening personal experiences, such as dealing with his father’s suicidal attempts and alcohol abuse, and his grandmother’s scary stories.

First-episode psychosis is characterized by psychotic symptoms prompting initial presentation for antipsychotic pharmacotherapy, leading to a formal diagnosis of schizophreniform disorder and, subsequently, schizophrenia, requiring treatment to stabilize symptoms [11]. Although our patient needed antipsychotic pharmacotherapy to stabilize his symptoms, he cannot be diagnosed with schizophrenia yet, mainly because of his lack of cognitive dysfunction. However, this case fits in to the schizophrenia spectrum and other psychotic disorders, which are defined by abnormalities in one or more of the following five domains: delusions, hallucinations, disorganized thinking, grossly disorganized or abnormal behavior, and negative symptoms [9].

There is a relationship between abuse and neglect in childhood and the intensity of psychotic symptoms. It is suggested that a history of childhood trauma is associated with increased sensitivity to stress [12]. In cases where psychological trauma in infancy leads to the development of psychotic symptoms, these symptoms become apparent at school age [13]. Moral harassment is one of the early life stressors most strongly associated with the presence of psychotic symptoms such as hallucinations and persecutory delusions [14].

The trauma model for psychosis is also grounded in biological changes such as (1) a reduction in brain structures; and (2) excessive activation of the hypothalamic–pituitary–adrenal axis leading to less efficient feedback in the hippocampus, causing a decrease in the number and sensitivity of glucocorticoid receptors. This decreased threshold favors the onset of psychiatric symptoms [15].

Although not well described in childhood, shared psychotic disorder arises between two entities: a pre-psychotic, in this case the child’s grandmother, and an individual susceptible to accepting the delirium of another, in this case our patient, a minor who experienced parental neglect throughout childhood and saw his grandmother as his only source of support [9]. The diagnosis of shared psychotic disorder is made only when the delirium is not due to the direct physiological effects of a substance or general medical condition [9].

The psychosis of two individuals can develop in a cultural environment that is isolated from others, where a partner responds to their partner’s delirium. The disorder usually starts with a difference in power and influence between the two, in which the one that induces the psychotic symptoms is the “real” psychotic and an individual with energetic and active character. However, the individual in whom psychotic symptoms are induced also plays an active role in their own psychosis, he engage in the delirium because of personal interests, such as the lure of profit, or the realization of a cherished dream [8]. In this case, the grandmother is the “real” psychotic, a person who is believed to have a respectable position within the family due to her “super power”, which grows in strength as more people believe her visions. The child is the induced, and it is possible that his benefits with the disorder is the fact that he receives more attention and love of his only caregiver.

Differential diagnosis is rarely a problem in these cases because the history of a close association with the primary case and the similarity between the delusions of the two individuals are specific to shared psychotic disorder [8]. In other mental disorders with psychotic symptoms, the individual has no intimate relationship with a dominant person who has a psychotic disorder and shares no similar delusional beliefs. If there is such a relationship, then the patient’s psychotic symptoms usually precede the onset of any shared delusions. In rare cases, an individual may present symptoms that appear to be a shared psychotic disorder, but their hallucination does not disappear when the individual is separated from the primary case. Another psychotic disorder diagnosis is likely to be considered once the shared psychotic disorder disappears with the separation of the two individuals [16]. In this case report, our patient’s symptoms ceased during his 3-day stay at the hospital, where the grandmother could not be present at all times. However, the child and his grandmother cannot be separated for significantly longer periods because the grandmother is his only caregiver.

The risk factors for the development of folie à deux are related to both the primary case and the induced patient’s characteristics. It is believed that this phenomenon presents multifactorial components in its determination, and both genetic and environmental components are involved in this process [17–20].

Individuals who develop induced psychotic disorder have a high risk for schizophrenia in their families, with a very high prevalence of schizophrenia in first-degree relatives (6.5–26.2%), similar to the prevalence seen in first-degree relatives of schizophrenic patients (5.0–16.9%) described in the literature. The prevalence of schizophrenia in families of both the primarily psychotic individual and the one who develops the psychosis is similar [17]. However, the participation of a genetic component does not exclude an environmental component; they are actually complementary [18]. Interpersonal relationships that are characterized by being close, lasting, and isolated from a social environment have also been identified as risk factors for the occurrence of folie à deux. This intimate interaction is an essential factor in the development of shared psychotic disorder. These environmental factors have been considered so important to the occurrence of the phenomenon that sometimes the simple separation of the individuals involved is enough to resolve the disorder [19].

An exact figure for the incidence and prevalence of folie à deux is not available, but cases from all over the world have been described and a recent review showed that such a phenomenon occurs more often in predisposed individuals in a context of social isolation. The incidence in married or common-law couples is equal to that in siblings, demonstrating that sex and age are not risk factors to the occurrence of the disorder, but only the fact that the pair cohabit [20].

## Conclusions

When treating patients presenting with psychotic symptoms with a history of neglect, who are in a close relationship with a person with a mental disorder, who lack social interaction, and who have a history of psychotic disorders in the family, treating physicians should be alert to the occurrence of folie à deux, because these are important proven risk factors for the development of this disorder. Therefore, early intervention to reduce the occurrence of child abuse and neglect should be considered in all patients with these characteristics.
